# Calcium Sets the Physiological Value of the Dominant Time Constant of Saturated Mouse Rod Photoresponse Recovery

**DOI:** 10.1371/journal.pone.0013025

**Published:** 2010-09-27

**Authors:** Frans Vinberg, Ari Koskelainen

**Affiliations:** Department of Biomedical Engineering and Computational Science (BECS), Aalto University School of Science and Technology, Espoo, Finland; Lund University, Sweden

## Abstract

**Background:**

The rate-limiting step that determines the dominant time constant (τ_D_) of mammalian rod photoresponse recovery is the deactivation of the active phosphodiesterase (PDE6). Physiologically relevant Ca^2+^-dependent mechanisms that would affect the PDE inactivation have not been identified. However, recently it has been shown that τ_D_ is modulated by background light in mouse rods.

**Methodology/Principal Findings:**

We used *ex vivo* ERG technique to record pharmacologically isolated photoreceptor responses (fast PIII component). We show a novel static effect of calcium on mouse rod phototransduction: Ca^2+^ shortens the dominant time constant (τ_D_) of saturated photoresponse recovery, i.e., when extracellular free Ca^2+^ is decreased from 1 mM to ∼25 nM, the τ_D_ is reversibly increased ∼1.5–2-fold.

**Conclusions:**

We conclude that the increase in τ_D_ during low Ca^2+^ treatment is not due to increased [cGMP], increased [Na^+^] or decreased [ATP] in rod outer segment (ROS). Also it cannot be due to protein translocation mechanisms. We suggest that a Ca^2+^-dependent mechanism controls the life time of active PDE.

## Introduction

In all eukaryotic cells a very steep gradient of Ca^2+^ exists across the plasma membrane. The intracellular calcium concentration ([Ca^2+^]_i_) is generally in the order of 100 nM while the extracellular Ca^2+^ level ([Ca^2+^]_o_) is above 1 mM. The steady-state concentration difference is set by delicate control of calcium influx through various types of calcium channels and by the extrusion of calcium ions through Na^+^/Ca^2+^ exchange (the Na^+^/Ca^2+^ exchangers, NCXs, or the Na^+^/Ca^2+^-K^+^ exchangers, NCKXs) and Ca^2+^-ATPases. This kind of dynamic control principle ensures that all the changes in the intracellular [Ca^2+^] are transient in nature, no matter whether the release of calcium is from intracellular stores or from the extracellular space, and this allows the wide use of calcium ion as internal transmitter in various types of signalling mechanisms.

In vertebrate photoreceptors in darkness, calcium enters the outer segment through cGMP-gated channels and is extruded by the NCKX. Light closes cGMP-gated channels, which reduces the influx of calcium while the calcium extrusion by the NCKX is continued, leading to a decrease in the intracellular calcium concentration. These changes in [Ca^2+^]_i_ during photoresponses are used as negative feedback signals in phototransduction. In mouse rods [Ca^2+^]_i_ is ∼250 nM in darkness and declines to ∼20–50 nM during bright illumination [1], [2]. This decrease in Ca^2+^ accelerates both the synthesis of cGMP [3], [4] and the inactivation of activated rhodopsin, R* [5], [6]. Further, in many photoreceptors, especially in most cones and in amphibian rods, the reduction of [Ca^2+^]_i_ is known to lower the affinity of the cGMP-gated channels to cGMP [7].

In addition to the dynamic effects described above, calcium ions also may have static effects on signalling. By “static effects” of calcium we refer to calcium-dependent mechanisms that participate in setting the operating point of a system (here phototransduction machinery) while moderate changes in the feedback signal (here [Ca^2+^]_i_) do not affect the output signal through the mechanisms of the static effect. In this study we demonstrate a novel effect of calcium on mouse rod phototransduction: calcium shortens the dominant time constant of saturated photoresponse recovery (τ_D_). Our data suggests that in mouse rods the intracellular calcium concentration needed to generate this effect may be below the values of [Ca^2+^]_i_ attained in bright light. Hence we call this effect of calcium on τ_D_ static. We show that τ_D_ is 1.5–2 times larger in very low free [Ca^2+^]_o_ (25 nM) compared to that in physiological [Ca^2+^]_o_ (1 mM). The effect of low external calcium was fully reversible, since the physiological value of τ_D_ was completely restored upon return to normal calcium even after long (>1 hour) exposures to low Ca^2+^. We show that the increase of τ_D_ in low Ca^2+^ was not due to high [cGMP]_i_ that results from the extensive activation of guanylate cyclase (GC) in low Ca^2+^. Also we show that neither highly increased ATP consumption nor increased intracellular sodium concentration can explain the increase of τ_D_ in low Ca^2+^. We suggest that in mouse rods there is a Ca^2+^ -dependent mechanism that controls the life-time of active PDE, and that a certain minimum level of intracellular calcium is needed in setting the physiological value of τ_D_.

## Results

### Determination of Dominant Time Constant by ERG from Isolated Retina Preparation

**Figure 1:**
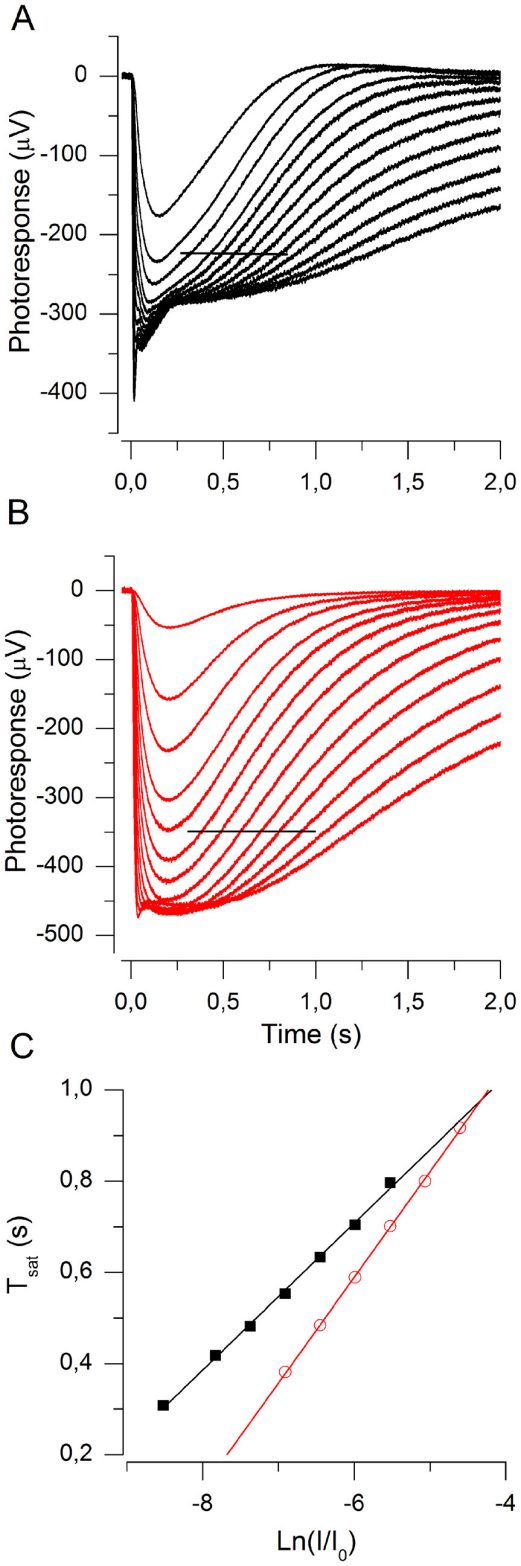
Determination of τ_D_ by the ERG method from isolated mouse retina. Recordings made from a retina perfused with bicarbonate-buffered Ringer at 37°C. (A) Family of responses to flashes of light in normal Ringer with 1 mM [Ca^2+^]. Flash strengths: 28, 87, 174, 348, 551, 874, 1390, 2200, 3480, 5510, 8740, 13900, 22000 Rh*. Data points used to determine τ_D_ ranged from 174 to 3480 Rh* (indicated by the horizontal line). (B) Response family in solution with 25 nM [Ca^2+^]_free_. Flash strengths were the same as in A and data points used to determine τ_D_ ranged from 874 to 8740 Rh* (indicated by the horizontal line). (C) Times for photoresponses to recover 20% (T_sat_) from saturation plotted as a function of the natural logarithm of normalized flash strength. Data points from A (▪, normal Ringer with 1 mM Ca^2+^) and B (red ○, low-Ca^2+^ solution, 25 nM Ca^2+^). τ_D_ was 161and 231 ms in normal Ringer and in low Ca^2+^, respectively.

Fig. 1A shows a photoresponse family recorded by the ERG technique across an isolated dark-adapted mouse retina perfused with bicarbonate-buffered (see Methods) Ringer solution at 37°C, when the ON-bipolar cell activity was suppressed by 50 µM DL-AP4. The saturated responses reflect the activity of both rods and cones [8], but the lower sensitivity and faster photoresponse kinetics of cones lead to a faster turn-off of the cone responses compared to rods. Further, the cone population is only 3% of all photoreceptors, leading to a low cone contribution to the ERG signal. Therefore the recovery phase of saturated ERG responses can be expected to reflect pure rod activity, as suggested by the common plateau level during rod saturation. The initial recovery of near-saturated and saturated responses closely follows the same slope with stimulus strengths covering the range from ca. 100 to 3500 Rh*, very similarly to those obtained by suction pipette recording [9]. Fig. 1C plots the times from flash (T_sat_) to the moment when the responses had recovered 20% from saturation (i.e. from the plateau level) as a function of stimulus strength in logarithmic scale. A linear regression line fitted to the data points (black squares) gives a dominant time constant τ_D_ of 161 ms (mean ± SEM: 166±12 ms, n = 4) which is comparable to τ_D_-values obtained for mouse rods by the double-flash ERG method *in vivo* [10] and by suction electrode recording from single rods, τ_D_ = ∼0.2 s [6], [11], [12].

### The Dominant Time Constant of Photoresponse Recovery Increases in Low Ca^2+^

Fig. 1B shows a corresponding response family after most of the extracellular calcium ions were removed by exchanging the perfusate to EGTA-buffered low Ca^2+^ solution with ∼25 nM free [Ca^2+^] (see Methods). When the retina was exposed to low Ca^2+^ solution, the saturated response amplitude showed a large initial increase (up to ∼4-fold). However, at 37°C these large responses could not be maintained long, and the saturated response amplitudes dropped quite fast (within ∼10 min) to the values ∼50–100% larger compared to normal Ca^2+^. After this the responses continued to decrease more slowly, being only somewhat larger than in normal Ca^2+^ after about 30 minutes exposure to low Ca^2+^. The responses in panel B were recorded between 22–32 minutes after the solution change to low Ca^2+^. Fig. 1C illustrates the T_sat_ data from these responses (red circles), showing that τ_D_ increased from 161 in normal Ringer to 231 ms in low Ca^2+^. A similar increase was observed in all four similar experiments at 37°C (166±12 in 1 mM Ca^2+^ to 251±15 ms in 25 nM Ca^2+^, n = 4). This novel Ca^2+^-dependent effect on τ_D_ is investigated in the following sections.

### Lowered Temperature Can Retain Large Responses in Low Calcium

**Figure 2:**
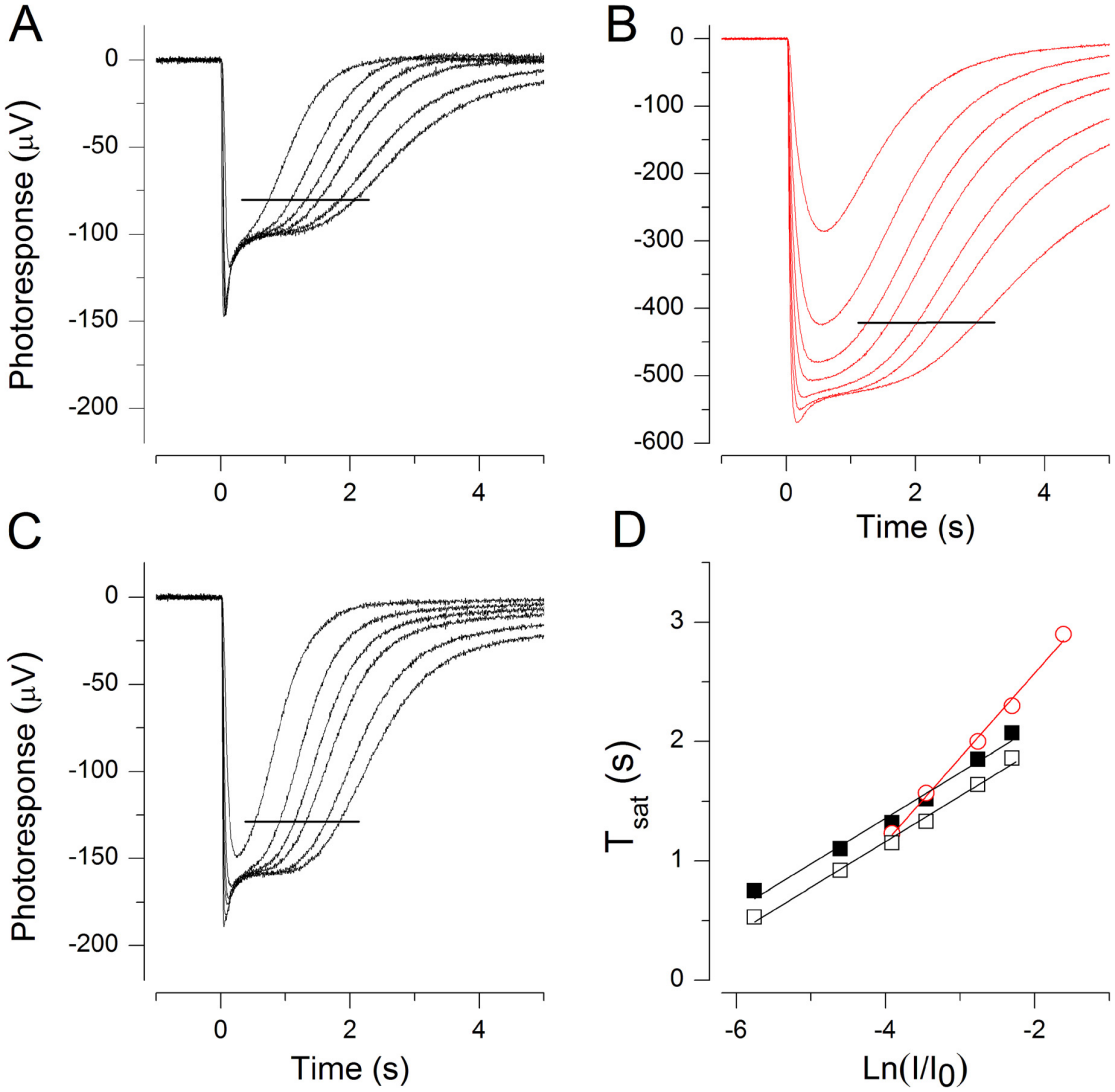
Dominant time constant τ_D_ is reversibly increased in low Ca^2+^. (A–C) Mouse ERG flash responses at 25°C in normal Ringer (A and C) and in 25 nM free Ca^2+^ (B). Flash strengths were 173, 548, 1100, 1730, 3460, 5480, (11 000, only in B) Rh*. Data used for τ_D_ determinations are indicated by horizontal lines. (D) Pepperberg plots extracted from A–C: τ_D_ was 383 ms before (▪) and 382 ms after (□) low Ca^2+^ (25 nM) exposure. In low Ca^2+^ τ_D_ was 710 ms (red ○).

The isolated mouse retina is a robust preparation that allows long-lasting treatments and experiments not easily attained with e.g. suction pipette recordings. However, the low external calcium concentration we used exerts a heavy metabolic load on photoreceptors: in these conditions intracellular calcium should go low enough to fully activate the calcium-controlled feedback mechanisms (guanylate cyclase through guanylate cyclase activating protein, GCAP, and rhodopsin through recoverin) in the rod outer segment even in darkness. The maximal guanylate cyclase activity leads to a substantial increase in [cGMP], and thus in the number of open cGMP-gated channels. Further, low external Ca^2+^ directly increases the single channel conductance of cGMP-gated channels [13], [14]. These metabolic factors together can most likely explain our observation that a steady-state could not be achieved in low calcium at 37°C, indicated by the continuous decay of saturated response amplitude. This decay, however, was largely absent at 25°C in HEPES-buffered Ringer. A further advantage of using HEPES-Ringer at 25°C is that, contrary to the bicarbonate-buffered Ringer at 37°C, the inner retinal contributions to the ERG can be very effectively pharmacologically removed (by 2 mM aspartate), as suggested by the simpler photoresponse shapes in Fig. 2A and C compared to the responses in bicarbonate-buffered Ringer at 37°C (Fig. 1A). We therefore chose to conduct the following experiments, tailored to investigate the Ca^2+^-dependent increase of τ_D_, in HEPES-buffered Ringer at 25°C.

Fig. 2 shows data from a typical experiment at 25°C. When the retina was exposed to low Ca^2+^ solution, the saturated response amplitude showed a very large initial increase that was followed by a decrease to a steady-state value ca. 5 times larger than the saturated photoresponse in 1 mM Ca^2+^ (measured at the plateau level). In other experiments the steady-state increase varied between 2 and 5-fold being 2.6-fold on average (n = 12). Despite of this large increase in the response amplitudes, the recordings in low Ca^2+^ were extremely stable. After ca. 10 minutes needed to reach a steady state in low Ca^2+^, the response amplitudes and kinetics remained virtually unchanged for at least 1 hour in most of the experiments.

**Figure 3:**
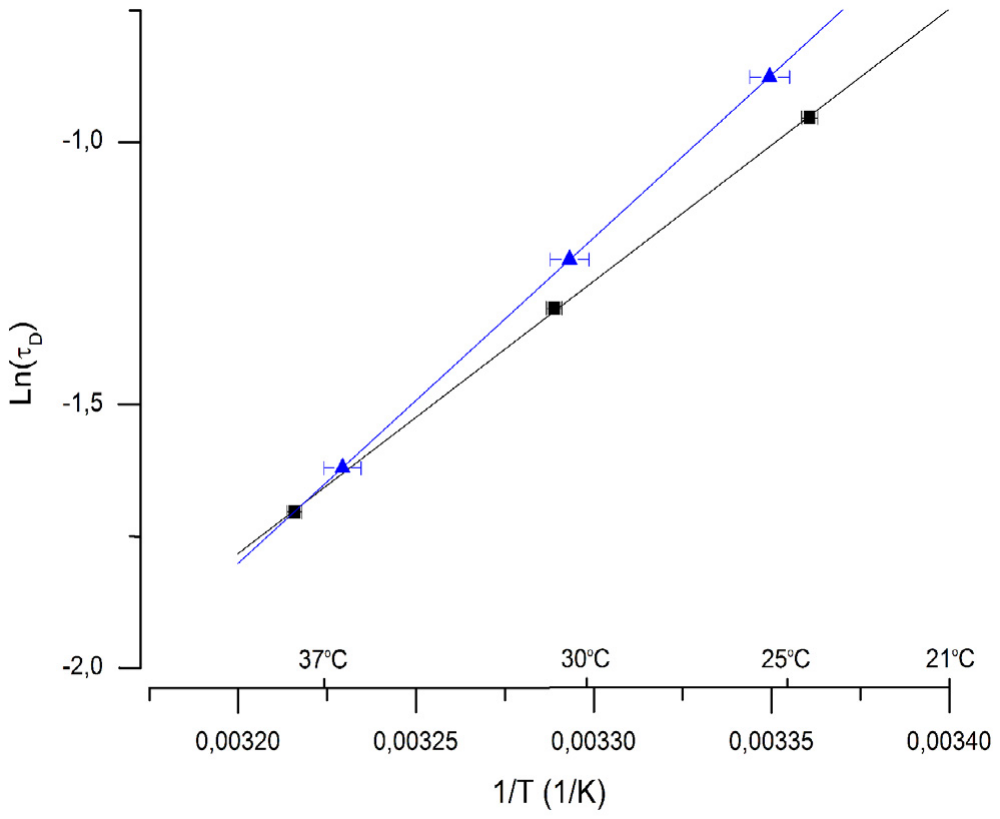
Temperature dependence of τ_D_. τ_D_ determined from the mouse rod response families at three different temperatures from two retinas. The natural logarithm of τ_D_ is plotted as a function of T^−1^. E_a_ was determined from the slope of the fitted straight lines (see Methods), giving 51.4 (blue ▴) and 43.0 (▪) kJ/mol, respectively.

The semi-saturated and saturated photoresponses of Fig. 2 to identical sets of 20 ms light flashes were recorded in 1 mM external calcium (A; before low Ca^2+^ exposure and C; after low Ca^2+^ exposure) and in 25 nM [Ca^2+^]_free_ (B). The collected T_sat_ data of these responses is presented in Fig. 2D. It shows that τ_D_ increased from 380 ms in 1 mM calcium to 710 ms in 25 nM Ca^2+^. The effects of low Ca^2+^ treatment on photoresponse kinetics, sensitivity and τ_D_ were always almost fully reversible, indicating that low Ca^2+^ treatment as such was not harmful to rods. As evident also in Fig. 2, the plateau amplitude often remained at a slightly larger level after the return to normal Ringer as compared to that before low-Ca^2+^ treatment. In this experiment the return to normal Ringer restored τ_D_ to its original value of 380 ms. In 12 experiments τ_D_ was (mean ± SEM) 342±28 ms in 1 mM calcium and 662±50 ms in 25 nM Ca^2+^, corresponding to 94% increase in τ_D_ when switched to low Ca^2+^. This value is somewhat larger than the observed 51% increase in τ_D_ at 37°C. To test whether the reason for this difference is that the molecular identity of the rate-limiting process of saturated photoresponse recovery changes (e.g. from PDE deactivation to rhodopsin deactivation) between 25°C and 37°C, we determined τ_D_ at three different temperatures (24–38°C) from two retinas in HEPES buffered Ringer. If the identity of the rate-limiting mechanism were different at 25°C and 37°C, and if these mechanisms had different activation energies, the slope of the Arrhenius plot would change between 25°C and 37°C. Fig. 3 shows that the temperature dependence followed accurately the simple Arrhenius equation (see Methods), suggesting that a single and same process is rate-limiting both at 25°C and 37°C. The activation energy of the process was about 47±4 kJ/mol (mean ± SEM, n = 2).

### The Effect of Cs^+^ on τ_D_

**Figure 4:**
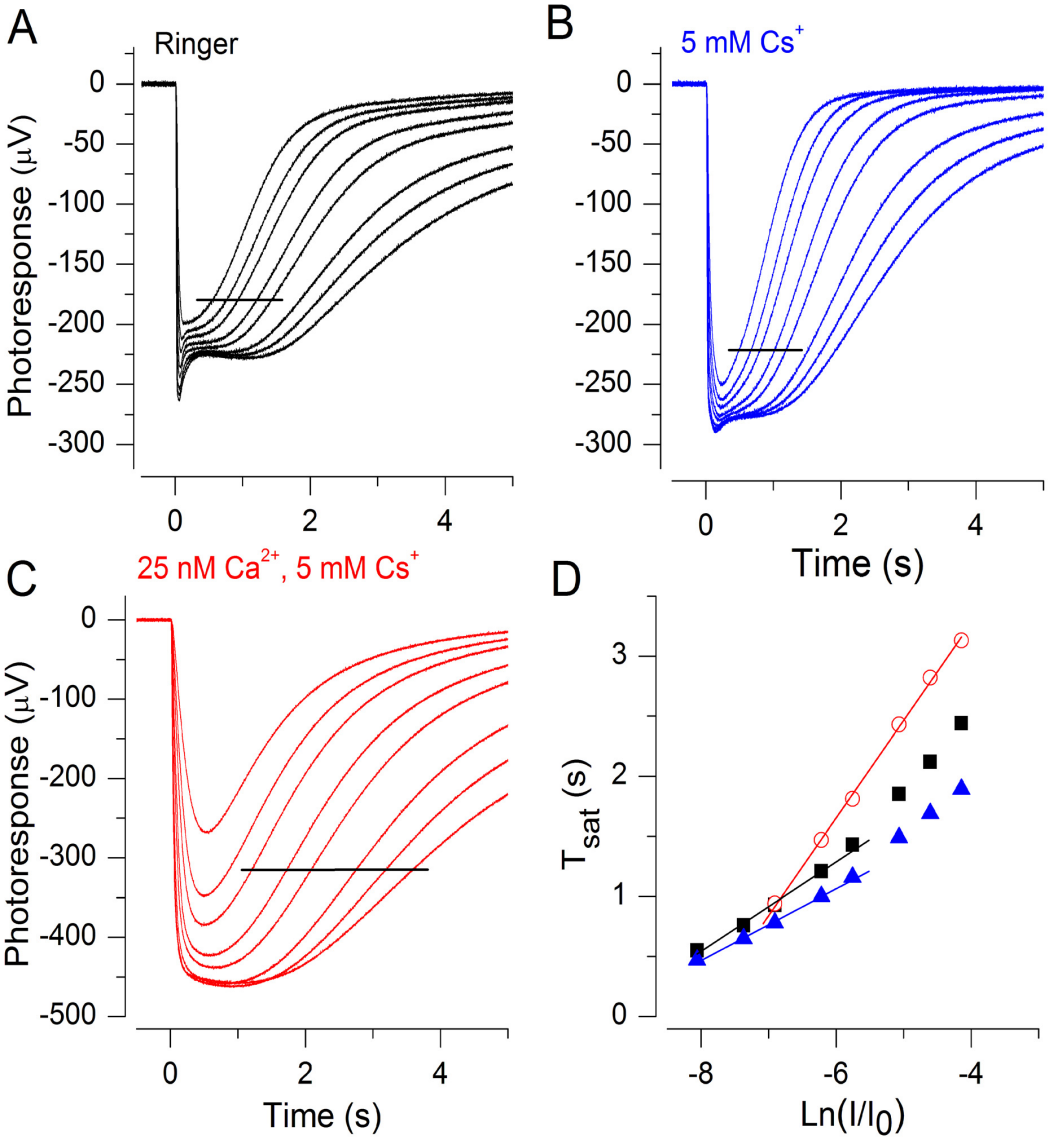
Effect of Cs^+^ on τ_D_. (A) Response family to flashes of light in HEPES-buffered Ringer containing 2 mM aspartate. Flash strengths: 140, 278, 441, 880, 1394, 2780, 4400, 7000 Rh*. (B) Response family to identical set of flashes as in A from the same retina after addition of 5 mM Cs^+^ to perfusion. (C) Identical stimulus set as in A and B when retina was exposed to low 25 nM free Ca^2+^ with 5 mM Cs^+^ present. Horizontal lines indicate the data points used in D. (D) Determination of τ_D_ from A (▪), B (blue ▴) and C (red ○).τ_D_ was 370, 300, 812 ms in Ringer, 5 mM Cs^+^ and in low Ca^2+^ with 5 mM Cs^+^, respectively.

The saturated rod responses include a fast negative peak (see Figs. 1A, 2A, C), the nose, which originates from the action of h and probably K_x_ channels in the rod inner segment [15]. This nose disappears in low extracellular Ca^2+^ (Figs. 1B and 2B), revealing a small cone component in the early phase of the saturated responses. To confirm that the increase in τ_D_ in low Ca^2+^ could not be due to the disappearance of the voltage-dependent nose component, we applied 3–5 mM Cs^+^, a blocker of hyperpolarization-activated h channels, which removes the nose-like behaviour in the saturated rod responses [15]. Fig. 4 shows the result from one such experiment. The introduction of 5 mM Cs^+^ accelerated the recovery rate of semi-saturated and saturated responses. This acceleration was qualitatively similar to that found in toad rod membrane voltage responses when 2 mM CsCl was introduced [16]. The accelerated photoresponse decay was accompanied by a decrease in τ_D_ from 370 ms to 300 ms with the addition of 5 mM Cs^+^. Similar or smaller decreases in τ_D_ were observed in the two other experiments of this kind. The reason for this small decrease in τ_D_ is not known, but the results convincingly show, that disappearance of h channel activity cannot be the reason for the increase in τ_D_ when switched to low Ca^2+^ solution. Figs. 4C and D show that when Ca^2+^ is lowered to 25 nM in the presence of Cs^+^, over 2-fold increase in τ_D_ is evident. This further confirms that although the voltage-dependent h channels in the rod inner segment might shape the saturated ERG photoresponses, their effect cannot explain the ∼2-fold increase in τ_D_ when [Ca^2+^]_o_ is lowered from 1 mM to 25 nM.

### Calcium Concentration Dependence of τ_D_

**Figure 5:**
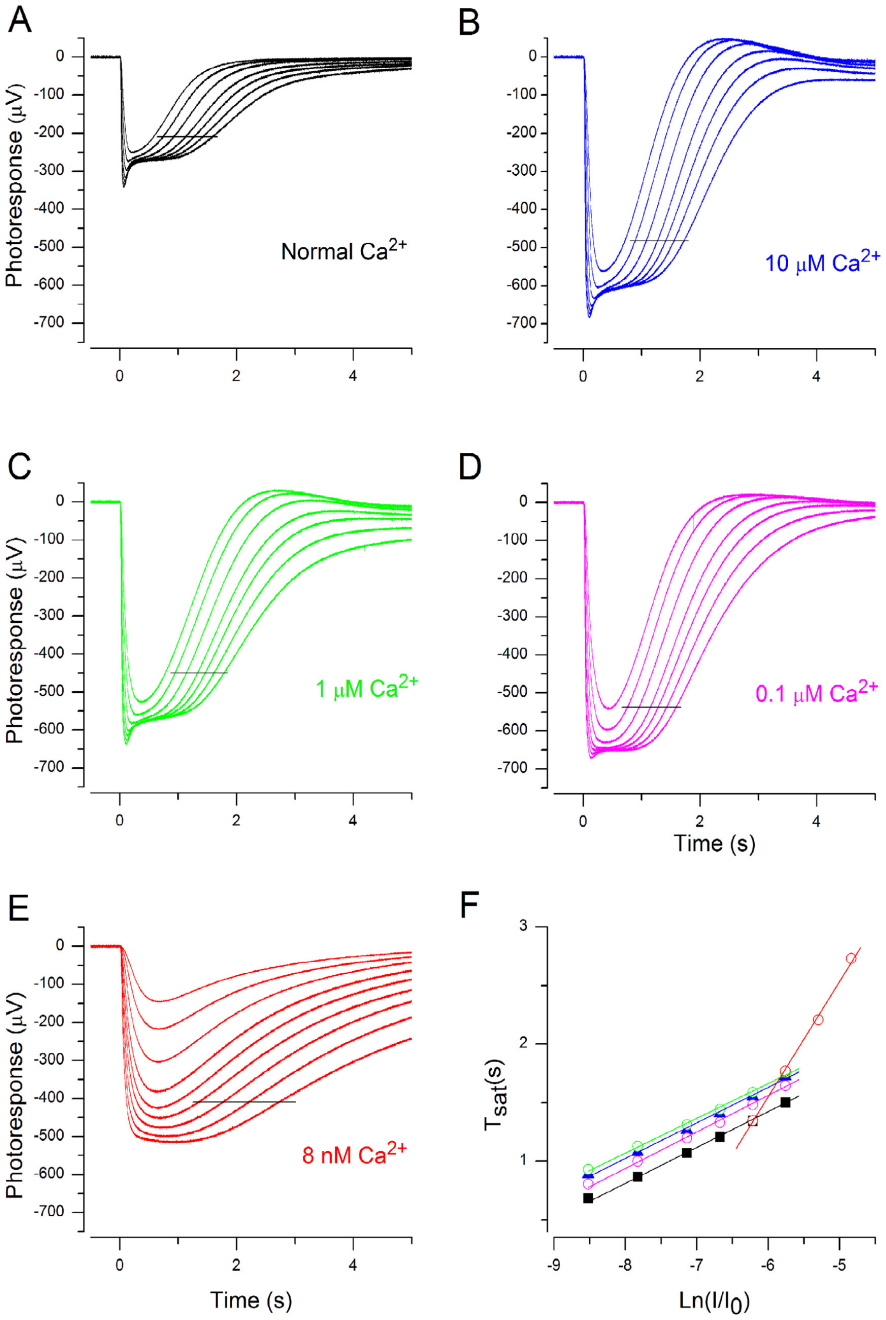
Effect of external [Ca^2+^] on τ_D_. Photoresponses at 1 mM (A), 10 µM (B), 1 µM (C), 0.1 µM (D), and 8 nM (E) free [Ca^2+^]_out_. Flash strengths: 87, 174, 348, 694, 1100, 1744, 2764, (4380, 6942, only in E) Rh*. (F) Dominant time constant determination from A–E. Data points used were between 174–2764 in A–D, and 1744–6942 Rh* in E, indicated by black horizontal lines in A–E. τ_D_ was 306, 303, 297, 311 and 983 ms in A, B, C, D and E, respectively.

We did three experiments in which we studied the dependence of τ_D_ on external [Ca^2+^]. In these experiments no lengthening of τ_D_ was observed until the free extracellular [Ca^2^] was reduced below 1 µM, although a large increase in saturated photoresponse amplitude was present already at 100 µM [Ca^2+^]_o_. Fig. 5 illustrates one such experiment in which τ_D_ was ∼300 ms in 1 mM Ca^2+^ and did not change significantly until the [Ca^2+^]_free_ in the perfusate was lowered to ∼10 nM. In the two other experiments τ_D_ was increased slightly (22 and 34%) at 100 nM free Ca^2+^, while the τ_D_ values at 10 nM [Ca^2+^]_free_ were 68 and 56% above those in normal Ringer. Although we do not know how much the declines in [Ca^2+^]_o_ reduce the intracellular calcium concentration at each [Ca^2+^]_o_, the large circulating current even at the lowest external calcium concentrations used in this study suggests, that there remains a substantial inward sodium gradient across the outer segment plasma membrane, that can drive the Na^+^/Ca^2+^-K^+^ exchanger to keep the intracellular calcium level below the [Ca^2+^]_o_. Therefore it is probable that the ∼two-fold increase in τ_D_ takes place only at intracellular calcium levels below those (20–50 nM, [1], [2]) attained in bright light.

### Increased cGMP Concentration Cannot Explain the Longer τ_D_ in Low Ca^2+^

**Figure 6:**
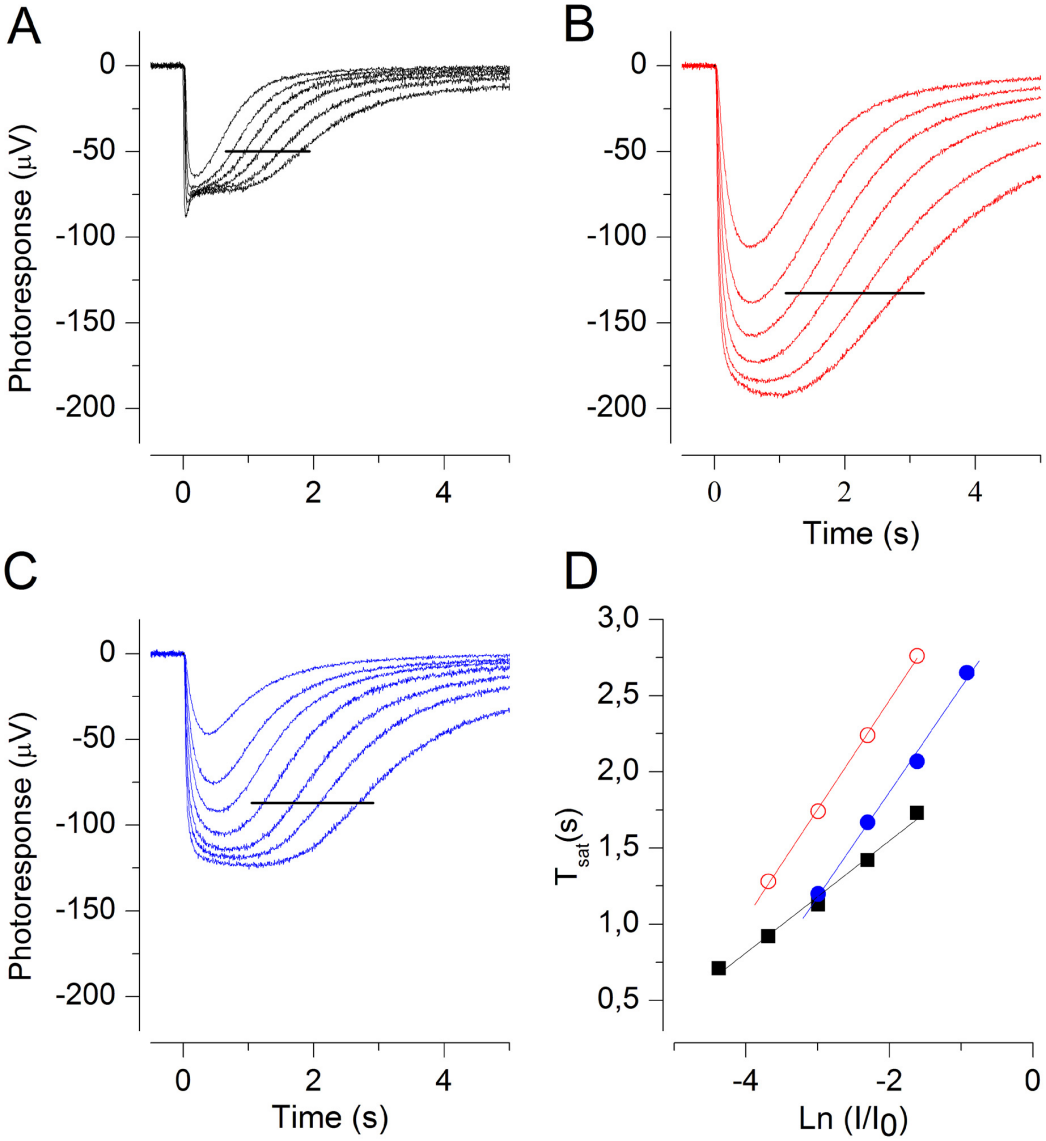
Background light does not prevent τ_D_ increase in low Ca^2+^. (A) Photoresponse family recorded in normal Ringer. Flash strengths: 425, 848, 1693, 3378, 6739, 13500 Rh*. Data points used to determine τ_D_ ranged from 848 to 13500 Rh* (indicated by the horizontal line). (B) Photoresponse family in 25 nM free [Ca^2+^]_o_. Flash strengths same as in A. Data points used to determine τ_D_ indicated by the horizontal line. (C) Photoresponse family in 25 nM free [Ca^2+^]_o_ during background illumination producing 640 Rh* s^−1^. Flash strengths: 425, 848, 1693, 3378, 6739, 13 500, 26800 Rh*. Data points used to determine τ_D_ ranged from 3378 to 26800 Rh* (indicated by the horizontal line). (D) Pepperberg plots extracted from A–C. τ_D_ increased from 360 ms (▪) to 660 ms (red ○) when retina was switched from normal Ringer to low Ca^2+^ solution. Background light producing 640 Rh* s^−1^ decreased τ_D_ to 600 ms (blue 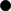).

One of the major consequences of lowered external calcium is the increase in [cGMP] in the ROS. To investigate whether elevated [cGMP] could explain the increase in τ_D_ in low Ca^2+^, we used background lights to reduce [cGMP] during low Ca^2+^ exposures. Fig. 6 illustrates one such experiment. Panel A shows a response family recorded in 1 mM calcium in darkness. Then the retina was exposed to 25 nM external Ca^2+^, and a response family was recorded after the responses had stabilized (panel B). Finally, response families were recorded under backgrounds of increasing intensity (range from 20 to 6400 Rh*/s per rod), when a steady-state had been achieved in each background. Fig. 6C presents a response family recorded in low Ca^2+^ under a background producing 640 Rh*/s per rod. Panel D collects the results of panels A–C, and for this retina τ_D_ was 360 ms in 1 mM Ca^2+^, increased to 660 ms in 25 nM Ca^2+^, and was slightly shorter, 600 ms, in 25 nM Ca^2+^ under the background. In this experiment response families were recorded under backgrounds producing 20, 64, 200, 640, 2000 and 6400 Rh* s^−1^ per rod. The corresponding τ_D_ values were 690, 680, 780, 600, 580 and 540 ms, respectively. Under the strongest background (6400 Rh* s^−1^ per rod) the saturated photoresponse amplitude was already smaller than in normal Ringer in darkness. Since lowered calcium is known to strongly increase the conductance of cGMP-gated channels in rods [13], [14], it is expected that the [cGMP] was much smaller under the strongest backgrounds in low Ca^2+^ compared to that in normal Ringer without background. With backgrounds exceeding 640 Rh* s^−1^ τ_D_ shortened somewhat with increasing background intensity, but remained significantly larger compared to that in normal Ca^2+^. These results suggest that the increase in τ_D_ during low Ca^2+^ exposures is not due to a direct or an indirect effect of elevated [cGMP].

Our second approach to investigate the possible effect of elevated cGMP concentration on τ_D_ was to increase [cGMP] concentration in the presence of normal Ringer by inhibiting PDE with IBMX. IBMX is known to boost photoresponse amplitudes and decelerate photoresponse kinetics as low Ca^2+^ treatment does, but to our knowledge, the effect of IBMX on τ_D_ has not been investigated before. We did two experiments in which we used two concentrations of IBMX, 30 and 100 µM. Instead of increasing, IBMX decreased τ_D_ slightly, being 270 ms in normal Ringer, and 260 and 250 ms at 30 and 100 µM IBMX, respectively. The same experimental protocol was repeated with another retina with similar results. Our results indicate that the level of steady-state [cGMP] is not important in determining τ_D_.

### Increased [Na^+^]_i_ or Decreased [ATP] in Outer Segment Cannot Explain the Longer τ_D_ in Low Ca^2+^

In dark-adapted mouse rods the major metabolic load in regard to ATP consumption comes from the extrusion of Na^+^ ions flowing into the ROS through the cGMP-gated channels [17]. In low Ca^2+^ this load is heavily raised: First, the guanylate cyclase is activated, leading to an increase in [cGMP] and thereby to an excess opening of cGMP-gated channels, and secondly, the conductance of single cGMP-gated channels is increased two- to threefold in low Ca^2+^ [13]. Consistently with this, Winkler and Riley have shown that in rat rods the Na^+^/K^+^ ATPase activity increases about 16-fold as the rat retina was switched from 2 mM Ca^2+^ to Ca^2+^-free media [18]. Thus, it seemed possible that in low Ca^2+^ the high ATP consumption might drop the ATP concentration in rods to a level low enough to slow down e.g. the phosphorylation processes involved in the phototransduction machinery, and thereby lead to an increase in τ_D_. Further, the increased cGMP-gated conductance in lowered external Ca^2+^ most likely leads to an increase in [Na^+^]_i_ that is assumed somehow to impair phototransduction [19]–[21].

**Figure 7:**
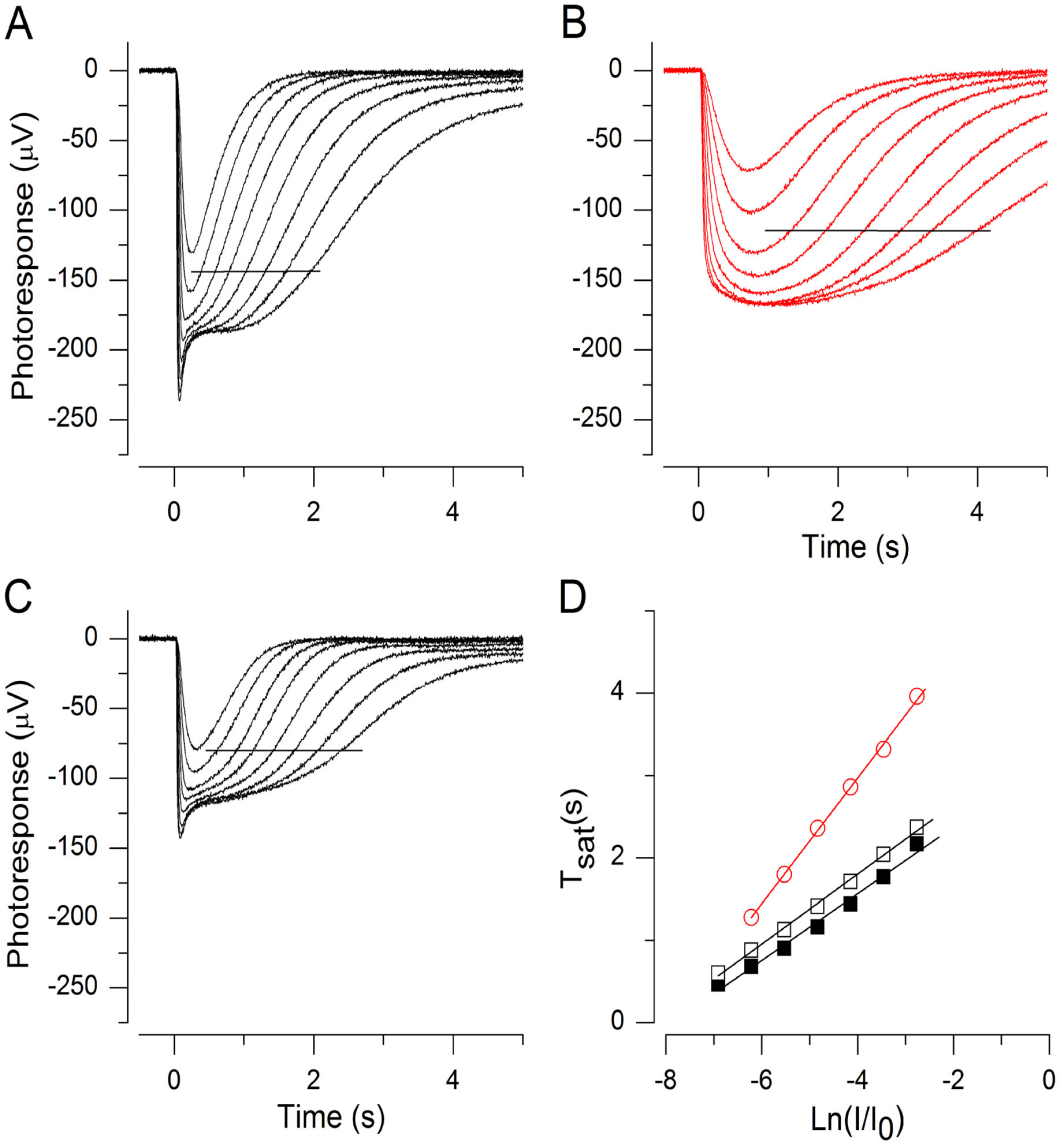
Lowered ATP/GTP and/or increased intracellular Na^+^ concentration does not explain the increase of τ_D_ in low Ca^2+^. (A) Response family in normal Ringer. Flash strengths: 52, 104, 207, 413, 824, 1643, 3280, 6540 Rh*. (B) Response family in solutions containing low 25 nM Ca^2+^ and low 8 mM Na^+^. Same flash strengths as in A. (C) Response family in normal Ringer after low Ca^2+^/Na^+^ exposure. (D) Pepperperg plots from A–C, data points indicated by horizontal line in A–C. τ_D_ was 400 ms before (▪) and 420 ms after (□) the low Ca^2+^/low Na^+^ exposure. It was increased to 760 ms in low Ca^2+^/low Na^+^ solution (red ○).

To test the possible effects of increased ATP consumption and increased [Na^+^]_i_ on τ_D_, we decreased the external sodium concentration from 140 mM to 8 mM in our low Ca^2+^ solution by replacing sodium with membrane impermeable choline. Fig. 7 shows the result. A control response family recorded in our normal Ringer is shown in panel A, while a corresponding response family recorded in 25 nM Ca^2+^ and 8 mM Na^+^ is shown in panel B. In low Ca^2+^-low Na^+^ the response amplitudes were not much reduced compared to those recorded in our normal Ringer, in spite of the fact that a large fraction of the current carriers through the cGMP-gated channels were removed. The responses remained very stable in these conditions. In this experiment τ_D_ was 400 ms in normal Ringer, increased to 760 ms in low Ca^2+^-low Na^+^ solution, and returned to 420 ms when Ringer was restored. Similar results were obtained in two other experiments conducted with the same protocol. The average increase in τ_D_ was (mean ± SEM) 174±44% when the perfusate was changed from our normal Ringer to low Ca^2+^-low Na^+^ Ringer. These results show that even in conditions where sodium accumulation inside the rods and the excessive ATP consumption are prevented, the exposure of rods to 25 nM Ca^2+^ results in a similar (or larger) increase in τ_D_ as observed in the presence of normal Na^+^.

## Discussion

### The Use of ERG Fast PIII Component in τ_D_ Determination

The canonical way to determine the dominant time constant of saturated photoresponse recovery is to suck either the outer or the inner segment of a photoreceptor into a tight-fitting suction pipette, and record the recovery kinetics of photoresponses to saturating light flashes of varying strength [22], [23]. To allow long and stable recordings under metabolically stressing conditions, and easy manipulation of the ionic environment around the entire photoreceptor cells, we instead used the isolated retina ERG technique for mouse rod τ_D_-determinations. The use of transretinal ERG for this purpose, however, brings about several possible sources of error that have to be carefully considered. Firstly, the transretinal ERG contains components from several types of neurons that temporally overlap the rod photoresponse recovery phase. These components should be removed by blocking all synaptic transmission from photoreceptors to bipolar and horizontal cells. In our low-Ca conditions the synaptic transmission was completely blocked presynaptically simply by the lack of calcium. In the presence of physiological external calcium the mGluR6 agonist L-AP4 effectively blocks signal transmission to ON-bipolar cells and thus completely removes the b-wave, leaving transmission to OFF-bipolar and horizontal cells intact. The ERG components from these latter cells are generally suppressed by the use of cocktails of different glutamate-receptor agonists and antagonists. Another approach is to perfuse the retina with the glutamate analog aspartate in mM concentrations to saturate all the glutamate binding sites in the various glutamate receptors. We observed that at 37°C in bicarbonate- and HEPES-buffered Ringer the combination of 50 µM DL-AP4 +10 µM NBQX was more effective than 2–5 mM aspartate in removing the second- and higher-order components, while at 25°C in HEPES-buffered Ringer both treatments were equally effective. Another robust component temporally overlapping with the recovery phase of fast PIII photoresponses is the glial component, or slow PIII, generated by the Müller cells in response to extracellular potassium changes due to photoreceptor activity. The slow PIII appears as a rounded component of same polarity as the fast PIII (see e.g. Fig. 1 in [24]) and can be removed by blocking the potassium channels of Müller cells by e.g. barium. To remove the slow PIII as completely as possible with minimally affecting the potassium channels in photoreceptors, we used high (10 mM) BaCl at the proximal side of the retina while perfusing the photoreceptor side with Ringer not including barium. With this approach it was possible to remove virtually all slow PIII at 25°C as indicated by the extending, horizontal plateau with increasing saturating stimulus strength. Under treatments described above the mouse fast PIII rod photoresponses look very much like the corresponding suction pipette current recordings from single mouse rods, and both recording methods give similar results for sensitivity and photoresponse kinetics. The major difference between these two methods is that in ERG the rate of photoresponse recovery may be modified by the voltage-dependent channels in the rod inner segment, while the suction pipette responses are not voltage-dependent in the physiological voltage range.

The determination of τ_D_ from saturated photoresponses by plotting the time photoreceptors remain in saturation as a function of logarithmic stimulus strength is, however, a very robust method. It is based on three key assumptions [22]: 1) Intracellular [cGMP] is solely determined by the rates of synthesis and hydrolysis of cGMP, and the slowest deactivation step after light stimulus is assumed to follow first-order kinetics. 2) Photoresponse recovery from saturation starts when [cGMP] has reached a certain criterion level specific to the experimental conditions. The criterion level of cGMP may be dissimilar in different experimental conditions, but it should not affect the τ_D_ value if the other conditions are met. 3) Guanylate cyclase is fully activated, i.e. constant, during the phase of photoresponse recovery that is used for τ_D_ determination. This condition is met when the intracellular calcium level has reached a value low enough to enable maximal guanylate cyclase activation by GCAPs, and the fulfilment of this condition is verified as the same slope of photoresponse recovery for responses to stimuli of different strengths when read at a certain amplitude criterion level. There is nothing in the assumptions that should make the “Pepperberg plots” voltage-dependent. This conclusion is supported e.g by the data of Baylor and Nunn (1986) [25]: When determined from their Fig. 2, the τ_D_ values are practically equivalent whether obtained from either the current or voltage responses simultaneously recorded from salamander rods. Therefore, transretinal ERG is expected to give the same τ_D_ values as the suction pipette method (or membrane potential recording) taken that components other than those of photoreceptor origin are carefully abolished. This is in agreement with our ERG data that gives τ_D_ = 166±12 ms (n = 4) in 1 mM external calcium at 37°C in the C57Bl/6N mice compared to τ_D_ = 168...246 ms obtained with single rod suction pipette recordings at the same temperature in darkness [6], [11], [12].

Mouse rod photoresponses appear to be sensitive to the Ringer composition perfusing the rods (see e.g. the supplemental figure in [26]). Therefore the reason for the observation that our ERG gives slightly smaller τ_D_ values than suction pipette recordings may lie in the minor differences in the perfusates used in these experiments, especially in the differences in pH buffers. In this study we observed that our (ERG) τ_D_ values were somewhat larger (τ_D_ = 194±5 ms, n = 4) at 37°C when determined in HEPES-buffered Ringer compared to those obtained in the presence of bicarbonate-buffered Ringer (τ_D_ = 166±12 ms, n = 4). The comparison of these values to those obtained with suction pipette recordings is not straightforward, since in most cases the filling solution of the suction pipette, where the rod outer segment is sucked in, has been buffered with HEPES while the solution perfusing the rod inner segment was buffered with bicarbonate. The τ_D_ values obtained by us with ERG and literature values obtained by suction pipette recordings match so closely that there seems to be no reason to believe that they might reflect different rate-limiting mechanisms.

### Effects of Low Ca^2+^ on Rods

Reducing intracellular Ca^2+^ to nM range has several effects on the phototransduction machinery in rod outer segments that potentially might cause the observed increase in τ_D_ in low calcium. The synthesis of cGMP by GC is strongly accelerated [3], [27], and the single channel conductance of cGMP-gated channels is increased [13], [14], [27]. Also the affinity of the cGMP-gated channel to cGMP is modulated by calcium [7], [28], but this effect seems to be negligible in mammalian rods [29]. These actions of low Ca^2+^ induce 1) elevation of intracellular [cGMP], 2) accumulation of Na^+^ inside rod outer segments [19]–[21] as well as 3) strongly increased ATP consumption [30], possibly leading to lowered intracellular ATP and GTP levels. In this study we focused ourselves to investigate whether these effects could explain the increased τ_D_ in low Ca^2+^.

The occupancy of the non-catalytic cGMP-binding sites of rod phosphodiesterase (PDE6) has been shown to control the GTPase activity of PDE, and the termination of PDE activity is slower when the non-catalytic binding sites are occupied and maximal when the sites are empty [31], [32]. The elevated [cGMP] in low calcium could, in principle, lead to full occupation of the non-catalytic binding sites and by that means increase τ_D_. In this study, however, we showed that a significant increase of τ_D_ was evident also when [cGMP]_i_ was strongly lowered (most probably well below the cGMP level in darkness in 1 mM external calcium) by background light during low Ca^2+^ exposure. This leads us to conclude that the occupation of PDE non-catalytic binding sites do not explain the increase of τ_D_ in low Ca^2+^.

Low Ca^2+^ supports very high inward Na^+^ current in ROS through cGMP channels. In toad the cGMP current increased transiently over 20-fold when switching from 1 mM Ca^2+^ to 0 Ca^2+^ plus 2 mM EGTA, but this dark current could not be maintained [19]. This suggests that Na^+^/K^+^ ATPase cannot maintain sodium gradient across plasma membrane in the presence of very high inward Na^+^ current, and that Na^+^ most probably is accumulated into the cell. Our experiments, however, suggest that elevated intracellular [Na^+^] itself does not increase τ_D_ in our low Ca^2+^ conditions. Firstly, the increase in τ_D_ is evident also in low Ca^2+^ Ringer containing only 8 mM Na^+^. Secondly, we did one experiment (not shown) in which we switched background light on just before introduction of low Ca^2+^ in order to suppress excessive cGMP-gated current. The intensity of the background light was chosen to keep the saturated response amplitude in low Ca^2+^ at a level approximately the same as in normal Ca^2+^ in darkness. Still τ_D_ increased in low Ca^2+^ (with background light) 67% from that in normal Ringer without background.

It has been calculated that the maintenance of ion gradients dominates the ATP consumption in darkness for mammalian rods even in normal Ca^2+^ [17]. Thus, the large circulating current in low Ca^2+^ must exert a very high metabolic load on mouse rods. In accordance with this, Winkler and Riley [18] have shown 16-fold increase in Na^+^/K^+^ ATPase activity in rat rods when exposed to low Ca^2+^. The high expenditure of ATP in low calcium may lead to the deficiency of ATP, which might retard phosphorylation processes, especially phosphorylation of active rhodopsin, and thus lengthen the lifetime of R*. Our experiments where we prevented the increase in the circulating current in low Ca^2+^ with 1) by lowering Na^+^ by more than one log unit or by 2) application of background light suggest that the larger τ_D_ in low Ca^2+^ is not due to the deficiency of ATP in mouse ROS.

One possible explanation for the increase in τ_D_ in low calcium could be the retardation of diffusion of the membrane proteins involved in phototransduction. This explanation, however, seems quite improbable: As a first approximation, instead of retardation one might expect acceleration of membrane protein diffusion, since the binding of the divalent calcium to the negative surface charges of disc membranes should rather increase than decrease the order parameter of the membranes, and thereby enhance the diffusion constants of the membrane proteins. Further evidence against this explanation comes from experiments on amphibian rods, where lowering the extracellular calcium level to nM range does not affect the diffusion constant of rhodopsin (Dr. V. Govardovskii, personal communication).

One further possibility for explaining the elevated τ_D_ in low Ca^2+^ is that the removal of calcium might induce translocation of a phototransduction protein or proteins between the outer and inner segments. To test this hypothesis we made fast solution change experiments (not shown) where we rapidly switched from 1 mM to 25 nM external calcium, recorded one response at a moment of about 30 s after the solution had changed at the retina, and immediately switched back to 1 mM calcium. These experiments showed that that τ_D_ had increased to its final value within 60 s after the solution change to low calcium. This approach ruled out the role of translocation of phototransduction proteins in τ_D_ increase.

### Target of Calcium-dependent τ_D_ Modulation

The seminal study by Krispel et al. (2006) [11] tied the molecular identity of the rate-limiting step in mouse rod photoresponse recovery in physiological conditions to the deactivation of PDE. The increase of τ_D_ observed in our low calcium experiments could, in principle, be explained by the deceleration of either PDE or rhodopsin deactivation. If the latter case were true, it would mean that in low calcium conditions PDE deactivation would be faster than rhodopsin deactivation, and now rhodopsin deactivation should be the rate-limiting step. This seems to be very unlikely since calcium-bound recoverin inhibits phosphorylation of rhodopsin [33], and light-induced decrease in [Ca^2+^]_i_ releases this inhibition and accelerates photoresponse recovery [34]. This is consistent with the observation that τ_D_ is the same in wild-type and recoverin knockout mice [34]. Therefore the most probable target behind the calcium-dependent modulation of τ_D_ seems to be PDE. The physiological significance of this novel mechanism may be that a certain amount of calcium is necessary in setting the operation point of the phototransduction machinery in regard to photoresponse recovery.

## Materials and Methods

### Preparation, Recording and Light Stimulation

#### Ethical Approval

The use and handling of all the animals in this study were in accordance with the Finnish Act on Animal Experimentation (62/2006) with guidelines of the Animal Experimentation Committee of Finland.

#### The ERG Experiments

Pigmented mice (C57Bl/6N) were dark-adapted overnight. The animals were sacrificed, the eyes were enucleated and bisected along the equator, and the retinas were detached in cooled Ringer under dim red light. The isolated retina was placed in a specimen holder [35], [36] with an active recording area of 1.2 mm or in later experiments 0.5 mm (diam.) at the flat-mounted central retina. The upper (photoreceptor) side was superfused with a constant flow (ca. 1.5 ml/min at 25°C and 3–5 ml/min at 37°C) of Ringer's solution. In the experiments at 25°C the Ringer contained (mM): Na^+^ 133.9, K^+^ 3.3, Mg^2+^ 2.0, Ca^2+^ 1.0; Cl^−^ 143.2, glucose, 10.0; EDTA, 0.01; Hepes, 12.0, buffered to pH 7.5 (at room temperature) with 5.8 mM NaOH. Sodium-L-aspartate (2 mM) was added to block synaptic transmission to second-order neurons. In the experiments at 37°C the Ringer contained (mM): Na^+^, 115.3; K^+^, 3.3; Mg^2+^, 2.0; Ca^2+^, 1.0; Cl^−^, 124.6; glucose, 10.0; EDTA, 0.01; HEPES 10.0; NaOH, 4.8 mM; NaHCO_3_, 20 mM. DL-2-Amino-4-phosphonobutyric acid (DL-AP4; 50* µ*M) was used to block synaptic signaling from photoreceptors to ON-bipolar cells. The Ringer was pre-heated to ca. 37°C and bubbled with a mixture of 95% O_2_ and 5% CO_2_ Leibovitz culture medium L-15 (Sigma), 0.72 mg/ml, was added to improve the viability of the retina. In addition in the experiments at 25°C, BaCl_2_ (10 mM) was added in the lower electrode space, from where it would diffuse to the retina to suppress glial currents by blocking potassium channels located mainly at the endfeet of Müller cells [24]. In experiments at 37°C 50 µM of BaCl_2_ was added to the perfusate, instead, because the 10 mM BaCl_2_ in the lower electrode space seemed to be less effective probably due to higher perfusion rate at 37°C. Pharmacological manipulations of the standard solution are explained below. The temperature was controlled by a heat exchanger below the specimen holder and monitored with a thermistor in the bath close to the retina.

#### Recording and Light Stimulation

The transretinal voltage changes (ERG) were recorded with two Ag/AgCl pellet electrodes, one in the subretinal space and the other in chloride solution connected to the perfusion Ringer through a porous plug. The DC signal was sampled at 200–10000 Hz with a voltage resolution of 0.25 µV.

The flashes used to produce short flash stimuli with homogeneous full-field illumination to the distal side of the retina were provided by a dual-beam optical system adapted from the setup used in [24]. In brief, 20 ms and later 2 ms light pulses were generated with a 543.5 nm HeNe laser (Melles Griot 05 LGR 173, 0.8 mW) or with a 532 nm laser (Power Technology IQ5C(532–100)L74, ∼130 mW) and a Compur (for 543.5 nm laser) or Oriel (model #76992, for 532 nm laser) shutter for both laser paths, the midpoint of the flash indicating the zero-time for the recordings. The Gaussian profile of the laser beam was flattened by conducting the beam through a light guide with mixing fibers. The uniformity of the beam at the level of the retina was confirmed with a small aperture photodiode. The light intensity of each source was controlled separately with calibrated neutral density filters and wedges.

The absolute intensity of the unattenuated laser beam (photons µm^−2^ s^−1^) incident on the retina (I_0_) was measured in each experiment with a calibrated photodiode (EG&G HUV-1000B; calibration by the National Standards Laboratory of Finland). The amount of isomerisations (Rh*) produced by the stimulating flash light in individual rods was calculated as described earlier [8].

### Chemicals and Pharmacological Manipulations

All the chemicals were purchased from Sigma-Aldrich Inc. In the lowest [Ca^2+^]_free_ (7.5–100 nM) solutions calcium was buffered with EGTA, and the free [Ca^2+^] was calculated using an “EGTA calculator” [37] taking into account 2 mM [Mg^2+^] present in our Ringer's solution. The low Na^+^ solution was prepared by substituting 132 mM [choline chloride] for NaCl, yielding 8 mM [Na^+^].

### Response Analysis

The dominant time constant τ_D_ was extracted by determining the time for semisaturated or saturated photoresponses to recover to a criterion level (T_sat_) which was chosen as 20–30% of the saturated photoresponse amplitude measured at plateau level after the nose [15] (see Fig. 1). The T_sat_ values were plotted against the natural logarithm of normalized flash intensity (Ln(I/I_0_)) and τ_D_ was then the slope of the fitted straight line.

The Arrhenius equation
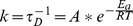
(1)was fitted to ln(τ_D_) data as a function of T^−1^ in the temperature range 24–38°C. There, k is the rate constant of the reaction, E_a_ is the activation energy of the reaction (J/mol), R is molar gas constant and T is the absolute temperature of the retina and A is a temperature-independent pre-exponential factor. E_a_ was calculated from the slope of the fitted straight line (E_a_  =  R_*_slope).
